# Distinct patterns of oligodendroglial dysfunction in neurodegenerative diseases

**DOI:** 10.1002/alz70855_104113

**Published:** 2025-12-24

**Authors:** Shelley L. Forrest, Madison Kane, Samantha Knott, Gabor G. Kovacs

**Affiliations:** ^1^ Tanz Centre for Research in Neurodegenerative Disease and Department of Laboratory Medicine and Pathobiology, University of Toronto, Toronto, ON, Canada; ^2^ Dementia Research Centre, Macquarie University, Sydney, NSW, Australia; ^3^ Tanz Centre for Research in Neurodegenerative Diseases, University of Toronto, Toronto, ON, Canada

## Abstract

**Background:**

Oligodendroglia are a major class of glial cell that play critical roles in myelination and neuronal support. Involvement of oligodendroglia is well‐known pathologically in frontotemporal lobar degeneration with tau (FTLD‐tau) and TDP‐43 (FTLD‐TDP) pathology, Alzheimer's disease (AD) and multiple system atrophy (MSA). However, their contribution to the pathogenesis of neurodegenerative disorders is underappreciated. This study investigated the impact of misfolded proteins including tau, TDP‐43, and a‐synuclein on the cellular characteristics of oligodendroglia and myelination.

**Method:**

82 cases were selected including controls (*n* = 5), AD (*n* = 10), FTLD‐tau (*n* = 38), FTLD‐TDP (*n* = 9), motor neuron disease (*n* = 10) and MSA (*n* = 10). Sections from two brain regions were examined: a pathological predilection site and minimally affected region, and immunostained for markers of oligodendroglia (tubulin polymerisation promoting protein, TPPP) and myelin (CNPase, PLP, MAG, MOG), and AT8, pTDP‐43 or a‐synuclein. Three cases of each group were selected for double‐labelled immunofluorescence to determine co‐localisation patterns. Density of oligodendroglial inclusions were assessed and oligodendroglial dysfunction measured by nuclear enlargement and fragmentation, assessment of TPPP‐immunostaining, co‐expression of TPPP with mis‐folded proteins, and assessment of myelin integrity.

**Result:**

Oligodendroglial inclusions were identified in all cases with the highest density found in MSA, FTLD‐tau subtypes and AD, followed by FTLD‐TDP and MND. Few oligodendroglial inclusions were found in the minimally affected region. In all groups, oligodendroglia with tau, TDP‐43 and a‐synuclein inclusions showed nuclear enlargement (*p* <0.001) and fragmentation (*p* <0.001) compared to controls and oligodendroglia without inclusions. Size of oligodendroglial nuclei with inclusions differed between groups and were most pronounced in MSA and cases with tau inclusions. TPPP mislocalised from the nucleus and cytoplasmic immunoreactivity differed between groups. Cytoplasmic TPPP‐immunoreactivity co‐localised with pathological protein inclusions. Myelin integrity was intact in all brain regions of control cases. Disruption of myelin integrity was prominent in all groups and was most pronounced in the pathological predilection area. Of all cases, those with AD, MSA and Pick's disease had the most severe disruption to myelin integrity.

**Conclusion:**

This study highlights the distinct cell‐intrinsic impact of mis‐folded protein pathology on oligodendroglia and identifies unique signatures of oligodendroglial dysfunction that are likely to adversely affect neuronal and axonal function.

